# Editorial to “The association between hyperuricemia and atrial fibrillation recurrence after catheter ablation”

**DOI:** 10.1002/joa3.13044

**Published:** 2024-04-18

**Authors:** Ugur Canpolat

**Affiliations:** ^1^ Department of Cardiology, Arrhythmia and Electrophysiology Unit Hacettepe University Faculty of Medicine Ankara Turkey

In the current issue of the *Journal of Arrhythmia*, Oseto et al.^1^ presented a retrospective study that included paroxysmal (*n* = 200) and persistent atrial fibrillation (*n* = 200) (PAF and PeAF) patients who underwent their first catheter ablation and evaluated the association of hyperuricemia (HU) with AF recurrence after catheter ablation. While the PAF patients underwent cryoballoon (CB)‐based pulmonary vein isolation (PVI), the PeAF patients underwent radiofrequency (RF)‐based PVI plus linear lines (roof and posterolateral mitral isthmus lines). Serum uric acid (SUA) levels were measured both 1 day before and 1, 3, and 6 months after the catheter ablation (the HU was defined as an SUA level of >7 mg/dL). As the ablation technique and strategy differed for both PAF and PeAF patients, the association of SUA/HU with AF recurrence after catheter ablation was assessed separately. The study results showed higher preablation SUA levels (6.5 ± 1.3 vs. 5.8 ± 1.3 mg/dL, *p* < .001) and HU rate (36% vs. 17%, *p* < .0001) in patients with PeAF than in patients with PAF. At 57 ± 24 months follow‐up, AF‐free survival in PAF patients was higher than in PeAF patients (84% vs. 58%, *p* < .0001), and post‐ablation HU (postprocedural 1st‐, 3rd‐, and 6th‐month samples) was significantly associated with AF recurrence only in PeAF patients. There was no association of peri‐procedural SUA levels and HU with AF recurrence after catheter ablation in PAF patients. The post‐ablation SUA reduction rate was higher in PeAF patients (*p* < .01). Reverse left atrial remodeling (reduction in left atrial diameter) 3 months after catheter ablation was also higher in PeAF patients without AF recurrence than in PeAF patients with AF recurrence. Although the authors recommended quitting alcohol intake before and after catheter ablation, there was no data about quitting rate or amount of alcohol intake per person and no specific dietary recommendation for patients with HU. While pre‐ and post‐ablation rates of SUA‐lowering medications were similar, there was a significant decrease in SUA level in PeAF after catheter ablation. No reasonable explanation was given for this reduction. No details are given in the paper about medications such as diuretics that may affect SUA levels before and after ablation. Furthermore, similar confounding factors for HU and AF recurrence including body mass index (BMI) and hypertension/heart failure rate were significantly higher in PeAF patients than in PAF patients. Since post‐ablation HU is associated with AF recurrence in PeAF patients, post‐ablation inflammatory marker levels could provide more valuable data to show the relationship with SUA. As the PeAF patients underwent RF ablation which was associated with more postprocedural tissue inflammation, edema, and necrosis, they could highlight the pathophysiological role of HU in AF recurrence. There was also no data about the atrial substrate and its relation with SUA levels and HU among patients with PeAF who underwent RF ablation using 3D‐electroanatomical mapping. Despite all the limitations mentioned above, this article demonstrates the importance of post‐ablation repeated measurement (*dynamicity of the biomarkers*) and persistent elevation of SUA level rather than a single SUA level elevation before the procedure in predicting AF recurrence after ablation.

Uric acid is a well‐known end‐product (by the action of xanthine oxidase) of purine metabolism in humans which is an anti‐oxidant at physiological levels and pro‐oxidant at hyperuricemic state (SUA level above 7 mg/dL in adult men or 6 mg/dL in adult women).^2^ Hyperuricemia (HU) is known as a risk factor for the onset and progression of AF via its role in inflammation and oxidative stress which result in atrial remodeling and fibrosis. Furthermore, HU is also associated with AF‐related risk factors including age, gender, adiposity/obesity, alcohol intake, hypertension, diabetes, coronary artery disease, chronic kidney disease, and obstructive sleep apnea.^2^ Thus, optimizing and controlling all those risk factors is the pillar of AF management to prevent both occurrence and progression in addition to SUA level and HU reduction.

Moreover, catheter ablation has a central role in AF management. Pulmonary vein isolation (PVI) is the gold standard approach in paroxysmal and persistent AF (PAF and PeAF) catheter ablation. However, because of the higher recurrence rates in PeAF after the PVI‐only strategy, additional ablation besides PVI is generally required. However, there is still a significant amount of AF recurrence after catheter ablation in both PAF and PeAF patients. Despite several periprocedural predictive indicators of AF recurrence after catheter ablation, the role of HU is controversial.^1^, ^3^ However, there were significant differences regarding the study sample sizes, AF subtypes, ablation techniques, risk factors, and follow‐up durations.

The SUA levels significantly differed between AF subtypes in a recent meta‐analysis (PeAF > PAF > new‐onset AF > no AF) which was consistent with the findings of Oseto et al. and confirms the relation of SUA level with the degree of atrial remodeling.^4^ The results of previous studies support that increased uric acid level plays an *active role* in the mechanism of atrial remodeling and AF rather than being an *innocent bystander*. Although the relation of preablation SUA levels alone with AF recurrence after catheter ablation was assessed in previous studies, Oseto et al.^1^ evaluated the association of serial SUA levels (preablation, post‐ablation 1st, 3rd, and 6th months) with AF recurrence after catheter ablation. Only post‐ablation SUA levels rather than preablation SUA levels were found as an independent predictor of AF recurrence after catheter ablation in PeAF patients. Oseto et al.^1^ showed that post‐ablation reduction of the HU rates in PeAF patients was independent of the SUA‐lowering medication rates. In parallel to this finding of Oseto et al., Aoyama et al.^5^ also showed a significant post‐ablation reduction in SUA levels independent from inflammatory parameters and kidney functions at 12‐month follow‐up. They also found that post‐ablation SUA level decline was significantly higher in patients with PeAF than in those with PAF (0.8 ± 1.0 vs. 0.2 ± 0.8 mg/dL, *p* < .001). However, the exact mechanism of such a reduction in SUA and HU was unclear. A significant reduction in post‐ablation SUA levels and HU rate parallel to post‐ablation reduction in left atrial diameter (*left atrial reverse remodeling*) among PeAF patients with no AF recurrence compared to PeAF patients with AF recurrence may explain the bidirectional association of SUA level/HU and atrial remodeling in the study by Oseta et al.

In conclusion, all those previous and current study findings highlight the potential of elevated SUA levels and HU as a useful indicator of atrial remodeling for stratifying risk in AF recurrence after catheter ablation, particularly in PeAF.

## CONFLICT OF INTEREST STATEMENT

The authors declare no conflicts of interest.
